# “Beyond-Zero-Sum” Range-Separated Local Hybrid Functional with Improved Dynamical Correlation

**DOI:** 10.1021/acs.jctc.5c00699

**Published:** 2025-07-18

**Authors:** Artur Wodyński, Martin Kaupp

**Affiliations:** Technische Universität Berlin, Institut für Chemie, 26524 Theoretische Chemie/Quantenchemie, Sekr. C7, Straße des 17. Juni 135, D-10623 Berlin, Germany

## Abstract

Recent work has shown that range-separated local hybrid (RSLH) functionals containing correction terms for strong correlation and delocalization errors, such as ωLH23tdE, allow a remarkable paradigm change in the context of the usual zero-sum game between delocalization and static correlation errors in the development of density functional approximations. In this work, we evaluate the modification of the dynamical correlation contribution for such strong correlation-corrected RSLHs (scRSLHs) and how it affects the performance of the large GMTKN55 database of main-group thermochemistry, kinetics, and noncovalent interactions. Replacing the B95c correlation in the previous functionals by a more flexible reoptimized B97c power-series expansion leads to substantial improvements. The ωLH25tdE scRSLH provides a GMTKN55 WTMAD-2 value of 2.64 kcal/mol in self-consistent calculations, when augmented by DFT-D4 corrections. This is the lowest for any rung 4 functional today. At the same time, ωLH25tdE retains the favorable performance for spin-restricted bond dissociation, the removal of unphysical spin contamination in certain open-shell transition-metal complexes, and the correct long-range asymptotic potential, resulting in excellent results for quasiparticle computations of ionization potentials, electron affinities, and band gaps.

## Graphical abstract

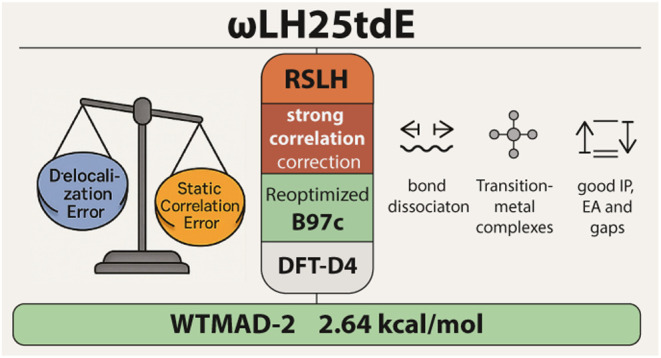

## 1 Introduction

Kohn-Sham density functional theory (KS-DFT) is the dominant workhorse in modern electronic structure theory in a wide variety of fields. A main objective in contemporary DFT research is to construct density functional approximations (DFAs) that simultaneously minimize static correlation errors (related to ”fractional-spin errors” ^1,2^ ) and delocalization errors (related to self-interaction and “fractional-charge” errors^3–6^ ). The vexing problem that often an improvement in one of these deteriorates the other has been called the “zero-sum game” of DFA development.^7,8^ In particular, a larger admixture of exact exchange (EXX) in global or range-separated hybrid functionals (“GHs” or "RSHs”) tends to reduce delocalization errors but worsen static correlation errors.

It has recently been shown^9,10^ that position-dependent EXX admixture in local hybrid functionals (LHs) and range-separated local hybrid functionals (RSLHs) offers a promising way to shift the playing ground (in mathematical terms “shift the Pareto front”) of the multiobjective optimization problem, that is, of the zero-sum game. In strong-correlation-corrected LHs (“scLHs”), we find that a local reduction of EXX admixture in regions where strong correlations are detected by suitable real-space functions (*q*_*AC*_ factors), even to locally negative admixtures, allows a strongly improved description of spin-restricted bond dissociation.^11–13^ This achievement could also be transferred to RSLHs in a somewhat more complicated way that also touches the range-separation procedure, leading to scRSLHs.^10,14^ Full long-range EXX admixture in RSLHs like ωLH22t allows a substantial reduction of delocalization errors and provides the correct long-range asymptotical exchange-correlation (XC) potential, thereby improving, for example, the description of charge-transfer excitations in time-dependent DFT (TDDFT) calculations and of ionization potentials (IPs), electron affinities (EAs), and band gaps via the frontier-orbital energies using Koopmans’ theorem.^15,16^ It is gratifying that scRSLH variants of ωLH22t like ωLH23tdE retain the latter advantages (a TDDFT implementation is ongoing work) while also improving on bond dissociation and generally reducing static correlation errors.^10^ These scRSLH functionals therefore feature currently among the most clear-cut escapes from the usual zero-sum game.^9^ Other attempts toward escaping the zero-sum game include the B13 ^17^ and KP16/B13 ^18^ functionals, as well as the mKP16 ^19^ extension to the latter. Notably, we found that additional terms in their local mixing functions (LMFs) governing the position-dependence of EXX admixture to address delocalization errors in abnormal open-shell regions help these scRSLHs not only to improve, for example, spin densities and hyperfine couplings in difficult transition-metal complexes^14^ but also to reduce the weighted mean absolute deviations (WTMAD-2 values)^10^ for the large GMTKN55 main-group test suite,^20^ which is typically assumed to cover largely systems with relatively weak correlations only. Meanwhile, improvements by scLHs have also been implemented and demonstrated to improve NMR chemical shifts and magnetizabilities in difficult cases, for example, for ozone and for certain transition-metal complexes.^21,22^ Note that the deep-neural-network functional DM21,^23^ which also provides a remarkable escape from the zero-sum game, should be viewed as an scRSLH given the input features handed to the deep-neural network underlying the entire functional. While we suspect that the same mechanisms allow its reduction of fractional-spin errors as we found for human-designed scLHs and scRSLHs, the completely black-box nature of DM21 prevents closer analysis.

Human-designed scLHs and scRSLHs so far all have added correction terms to a so-called t-LMF^24–26^ (a scaled ratio between von-Weizsäcker and KS kinetic-energy densities, $g^{t-\mathrm{LMF}}\left(r\right)=a\frac{\tau_{w}\left(r\right)}{\tau \left(r\right)}$ ) governing the position-dependent EXX admixture. Given that we know only few exact constraints on the LMF of an LH, and none in the valence space, we have recently constructed a so-called “n-LMF” as a small and shallow neural network.^27^ In spite of the relatively small training data set, the resulting LH24n-B95 and LH24n functionals exhibited a substantial reduction of WTMAD-2 values for GMTKN55 over the related LH20t functional with a t-LMF, suggesting that data-driven improvements of LMFs may be a viable pathway toward constructing better LHs and RSLHs. Indeed, some of the improvements could even be transferred from the “hybrid” rung 4 of Perdew’s ladder hierarchy of functionals to the “double hybrid” rung 5, with promising preliminary results reported for “range-separated local double hybrids”.^28^ One extremely favorable feature of n-LMFs is that they largely suppress the so-called “gauge problem” of LHs and RSLHs arising from the ambiguity of exchange-energy densities, without requiring the addition of a calibration function (CF) to deal with, in particular, noncovalent interactions.

However, first attempts to incorporate sc correction terms into n-LMFs so far have been unsuccessful, in part due to the very large EXX admixture of n-LMFs in the bond region, which makes it more onerous to reduce it locally in strong correlation regions. Optimizing machine-learned LMFs with sc terms is an ongoing research area in our group. Here, we want to instead exploit another observation made in ref 27 to advance the field of scRSLHs beyond the zero-sum game even further: our previous generation of scLHs and scRSLHs all inherited a B95c dynamical correlation contribution^29^ from the underlying LH20t^30^ and ωLH22t,^15^ respectively. We found that replacing this B95c functional by a more flexible power-series expansion B97c-type correlation functional,^31^ and training its linear parameters on a larger database like GMTKN55, provides substantial improvements, particularly for noncovalent interactions (NCIs) in conjunction with DFT-D4-style dispersion corrections. That is, LH24n-D4 with B97c correlation improves the WTMAD-2 value over LH24n-B95 with the original B95c correlation functional of LH20t from 3.49 to 3.10 kcal/mol.^27^ Here, we investigate whether such an improvement can also be harnessed when using a data-driven B97c expansion to improve upon an scRSLH such as ωLH23tdE. This leads to the ωLH25tdE functional. When augmented by DFT-D4 corrections,^32–34^ it provides the lowest WTMAD-2 value, as well as the lowest mean absolute deviations for the large W4-11RE reaction-energy database, of any rung 4 functional to date while retaining the small static correlation and delocalization errors of ωLH23tdE.

## 2 Theory

### 2.1 Reviewing the ωLH23tdE Functional

The ωLH23tdE scRSLH can be written as^10^

1 $$E_{\mathrm{XC}}^{\mathrm{scRSLH}}=E_{X}^{\mathrm{ex}}+\int 2q_{\mathrm{AC}}\left(r\right)\left(\left(1-g\left(r\right)\right)\cdot \left(1-a_{d}\left(r\right)\right)\sum_{\sigma }\left(\Delta e_{\mathrm{SR},\sigma }\left(r\right)+f_{\mathrm{FR}}\left(r\right)\Delta e_{\mathrm{LR},\sigma }\left(r\right)\right)+e_{C}^{B95}\left(r\right)\right)dr$$

where

2 $$\Delta e_{\mathrm{SR},\sigma }\left(r\right)=e_{X,\sigma }^{\mathrm{PBE},\mathrm{SR},\omega }\left(r\right)+G_{\sigma }^{\mathrm{pig}2}\left(r\right)-e_{X,\sigma }^{\mathrm{ex},\mathrm{SR},\omega }\left(r\right)$$

defines the short-range local hybrid correction to exact exchange, based on the difference between semilocal (PBE) and exact exchange-energy densities. As ωLH23tdE is based on a t-LMF, we cannot count on an automatic suppression of the gauge problem (see Introduction) and use the second-order partial integration CF, *G*_σ_^*pig*2^(**r**),^35^ to minimize gauge artifacts, in particular regarding NCIs. We also define the long-range counterpart:

3 $$\Delta e_{\mathrm{LR},\sigma }\left(r\right)=e_{X,\sigma }^{\mathrm{PBE},\mathrm{LR},\omega }\left(r\right)-e_{X,\sigma }^{\mathrm{ex},\mathrm{LR},\omega }\left(r\right)$$

The sc correction to the LMF,^10,13^

4 $$q_{\mathrm{AC}}^{\mathrm{erf}}\left(r\right)=0.5+\frac{\mathrm{erf}\left(bz^{d}\left(r\right)\right)}{2}$$

where *b* is an adjustable enhancement factor, serves to recover kinetic-energy contributions to strong correlations from a local adiabatic connection akin to the B13 ^17^ and KP16/B13 ^18^ real-space models. In ωLH23tdE, the detection of spatial regions with strong correlations within *q*_*AC*_^erf^(**r**) is based on a damped real-space ratio of semilocal and exact exchange-energy densities,

5 $$z^{d}\left(r\right)=\max\left(z\left(r\right),0\right)\cdot \mathrm{erf}\left(12.0\cdot \max\left(z\left(r\right)-c,0\right)\right)$$

6 $$z\left(r\right)=\frac{e_{X,\alpha }^{\mathrm{sl}}\left(r\right)+e_{X,\beta }^{\mathrm{sl}}\left(r\right)}{e_{X,\alpha }^{\mathrm{ex}}\left(r\right)+e_{X,\beta }^{\mathrm{ex}}\left(r\right)}-1$$

where *c* is an adjustable damping factor. We note in passing that *q*_AC_^erf^(**r**) in eq 1 connects adiabatically not only the middle nondynamical correlation terms but also the semilocal *e*_*C*_^*B*95^(**r**) dynamical correlation energy density.

Since static correlation has a distinct long-range character, while the nondynamical correlation term of the underlying ωLH22t RSLH contains only short-range exchange-energy densities, a long-range correction (eq 3) is added to the scRSLH. It is governed by the real-space switching function *f*_*FR*_(**r**), which depends on the same *z*(**r**) factor as *q*_*AC*_(**r**), where *q*_*AC*_(**r**) goes from 0.5 in the weak-correlation limit to 1.0 for maximal sc correction and *f*_*FR*_(**r**) goes from 0.0 to 1.0 (see ref 10 for further explanations).

ωLH23tdE contains another correction to the t-LMF to reduce delocalization errors in abnormal open-shell regions (“DEC correction term”^10^),

7 $$a_{d}\left(r\right)=\left(\sqrt{\zeta^{2}\left(r\right)+\delta }-\sqrt{\delta }\right)\cdot \mathrm{erf}\left(h\cdot \max\left(z\left(r\right),0\right)\right)$$

inspired by the PSTS functional.^36^ We reuse here the function *z*(**r**) in a different role, and *h* is an adjustable enhancement factor. That is, this ratio can be involved in DEC corrections as well as static correlation errors. We presume that smaller values of *z*(**r**) may reflect open-shell spatial regions with delocalization errors, while larger values are more indicative of sc effects. This assumption is purely empirical and based on previous observations that the B13 or KP16 functionals, which use essentially “undamped” sc factors, perform well for strong correlation cases but somewhat less well in weakly correlated situations, and we have made similar observations for scLHs without damping. Note that self-interaction errors for (semi)­local exchange functionals and the ability of these functionals to partly simulate left–right correlation in bonds are closely connected, and a full disentanglement does not seem to be possible.^37,38^ Equation 1 splits these contributions in a heuristic way. ζ­(**r**) represents the spin polarization.

ωLH23tdE had retained all parameters of the underlying ωLH22t RSLH, which had been optimized in equally weighted form for the rather limited W4-08 atomization energy^39^ and BH76 reaction-barrier^40,41^ test sets. Only the sc- and DEC correction terms were added to this in ωLH23tdE. This obviously suggests room for improvements in constructing and training a more flexible scRSLH model.

### 2.2 Data-Driven Improvements of Dynamical Correlation, the ωLH25tdE Functional

We had observed previously for some LHs and RSLHs that the B95c correlation may not be sufficiently flexible to allow the optimum description of NCIs and generally weaker interactions when using D3 or D4 dispersion corrections. Often, the improvements on, for example, the NCI subcategories of GMTKN55 upon adding the dispersion terms turned out to be relatively small, for example, for RSLHs and scRSLHs or for the LH23pt LH with a modified pt-LMF.^42^ In ref 27, we therefore introduced a more flexible B97c-style power-series expansion in LH24n and found that the non-self-consistent optimization of its linear parameters for the full GMTKN55 suite together with the D4 parameters improved in particular on the NCI subcategory compared to LH24n-B95, which had retained the original B95c parametrization of LH20t. For ωLH25tdE, we therefore replace the B95c energy density in eq 1 by a corresponding reoptimized B97c energy density. The B97c-type correlation functional with its opposite- and same-spin energy densities reads

8 $$e_{B97c}\left(r\right)=e_{B97c}^{\mathrm{opp}}\left(r\right)+\sum_{\sigma }e_{B97c}^{\sigma \sigma }\left(r\right)$$

9 $$e_{B97c}^{\mathrm{opp}}\left(r\right)=\sum_{i=0,m}d_{\mathrm{opp},i}{\left(\frac{c_{\mathrm{opp}}\left(\chi_{\alpha }^{2}\left(r\right)+\chi_{\beta }^{2}\left(r\right)\right)}{1+c_{\mathrm{opp}}\left(\chi_{\alpha }^{2}\left(r\right)+\chi_{\beta }^{2}\left(r\right)\right)}\right)}^{i}\cdot e_{c,\mathrm{opp}}^{\mathrm{UEG}}\left(r\right)$$

10 $$e_{B97c}^{\sigma \sigma }\left(r\right)=\sum_{i=0,m}d_{\sigma \sigma ,i}{\left(\frac{c_{\sigma \sigma }\left(\chi_{\sigma }^{2}\left(r\right)\right)}{1+c_{\sigma \sigma }\left(\chi_{\sigma }^{2}\left(r\right)\right)}\right)}^{i}\cdot \alpha_{\sigma }\left(r\right)\cdot e_{c,\sigma \sigma }^{\mathrm{UEG}}\left(r\right)$$

where *d*_opp,i_ and *d*_*σσ*,i_ are linear parameters in the power-series expansion up to order *m* and *c*_opp_ and *c*_*σσ*_ are nonlinear parameters. *e*_c,opp_^UEG^ and *e*_c,*σσ*_^UEG^ are homogeneous electron–gas correlation potential-energy densities. The χ_σ_ functions are gradient-based enhancement factors, while α_σ_ is a meta-GGA same-spin self-correlation correction based on kinetic-energy densities. See ref 27 for more details. We note that B95c can be considered a special case of B97c for *m* = 2, when neglecting certain terms. This implies that B97c is a more flexible extension of B95c.

## 3 Computational Details

The training of the linear parameters of ωLH25tdE (up to *m* = 3 of B97c in eqs 9 and 10) was performed post-SCF with the in-house Python code B97opt.^43^ The nonlinear B97c parameters *c*_opp_ and *c*_*σσ*_ (see eqs 9 and 10), as well as parameters *b*, *c* in *q*_AC_^erf^(**r**) (see eqs 4 and 5), range-separation parameter ω, and parameter *h* in *a*_*d*_(**r**) (see eq 7) were retained from ωLH23tdE.^10^ Similarly, the parameters of the pig2 CF were taken from ωLH23tdE, as the middle nondynamical correlation term did not change significantly from that functional. As done in other cases, we additionally rescaled the LMF (the product of t-LMF and DEC term) by a linear prefactor *e*, leading to

$$g^{c}\left(r\right)=1-e\cdot \left(1-g^{t-\mathrm{LMF}}\left(r\right)\right)\left(1-a_{d}\left(r\right)\right)$$

while retaining the linear parameter of *g* ^t–LMF^(**r**) from ωLH23tdE.

The GMTKN55 data used during training were obtained from ωLH23tdE orbitals in Turbomole (local developers’ version based on release 7.8) using integration *gridsize* 4 and def2-QZVP^44^ basis sets, with diffuse functions added for selected subsets, as described in ref 20 .

The linear (*s*_8_) and nonlinear (*a*_1_ and *a*_2_) parameters of the D4 dispersion correction were optimized alongside the linear parameters of the functional by minimizing the WTMAD-2 metric of the GMTKN55 benchmark suite. To estimate the transferability of the trained parameters, we also split the subsets of the GMTKN55 suite into smaller random sets. Then, we used these smaller parts for optimization and the remainder of the test suite for validation. The final overall 19 parameters of ωLH25tdE-D4 (including 9 introduced in this work) are given in Table S1 in Supporting Information, which also contains the three parameters of the D4 corrections.

Self-consistent computations were done using our Turbomole developers’ version. Calculations for GMTKN55 again employed def2-QZVP^44^ in the same manner as for the generation of post-SCF training data. The Turbomole *gridsize* was set to m4, which is also consistent with common practice. Further validation of ωLH25tdE was performed on real-world transition-metal organometallic reaction energies using MOR41 ^45^ closed-shell and ROST61 ^46^ for open-shell complexes, as well as on barrier heights using a modified MOBH35 ^47–50^ subset, MOBH28.^51^ These additional calculations employed def2-QZVPP^44^ basis sets, along with Stuttgart–Dresden scalar-relativistic pseudopotentials for 4d and 5d transition-metal atoms,^52^ and *gridsize* m5.

The calculations of asymptotic energies of the DISS10 set of spin-restricted diatomic bond-dissociation curves as a measure of strong correlation errors^12^ were performed with *gridsize* m3 and def2-QZVPPD orbital basis sets. Full dissociation curves of the noble-gas radical cation dimers (Ar_2_^+^ and Ne_2_^+^) as a measure of delocalization error were calculated also with the def2-QZVPPD basis set and *gridsize* 4, additionally selecting the *diffuse* 2 option in grid construction.

Calculation of HFCs of the prototypical MnO_3_ complex known to be sensitive to spin contamination uses the scalar relativistic X2C Hamiltonian^53,54^ and the corresponding picture-change-corrected HFC operator.^55^ These calculations employed the fully uncontracted versions of the NMR_9s7p4d^56^ and IGLO-III^57^ basis sets with *gridsize* 3 to be consistent with previous works.^14,56^

Computations of the EAs of second- and third-period p-block atoms from the highest-molecular-orbital (HOMO) energies of the anions^58^ have been performed using *gridsize* 7 and the aug-pc-∞ basis set provided in that work, where an uncontracted aug-pc-4 basis was extended by more and more diffuse s- and p-functions in a geometric progression with a factor √10 until the smallest exponent was below 10^–10^.^58^ We include only those anions, where the extra electron is bound in the reference data set, as otherwise results tend to reflect basis-set space rather than any physical observable (i.e., noble-gas atoms as well as Be, Mg, and N are excluded). The hydride ion has also been excluded for reasons discussed in ref 10. We will look at the negative of the HOMO energies of the anions using Koopmans’ theorem.^59^ Note that several of the anions in that test set^58^ exhibit open-shell character (B, C, O, Al, Si, P, S). Here, the results are based on the extension of Koopmans’ theorem to unrestricted KS theory by Gritsenko and Baerends.^60,61^ That is, for anions with a less than half-filled shell (B, C, Al, Si), the HOMO is the highest filled α-orbital, and for the other cases (O, P, S), it is the highest filled β-orbital.^16^

For the various organic chromophore test sets, we also used the same basis sets as employed in the original works. Unless noted otherwise, HOMO energies of the neutral molecules have been used to compute IPs, the lowest-unoccupied molecular orbital (LUMO) energies to obtain the EAs, and fundamental gaps were extracted from the difference between LUMO and HOMO energies. For the oligoacenes from *n* = 1 (benzene) through *n* = 6 (hexacene),^62^ *gridsize* 3 and cc-pVTZ^63^ basis sets have been used. The acceptor molecules from ref 64 . have also been computed using *gridsize* 3, with the same point-group symmetries as in that previous work and with aug-cc-pVTZ^63^ basis sets.

Two-electron integrals required for EXX energy densities were computed using a seminumerical integration approach,^65–68^ with standard screening settings provided by Turbomole. In most cases, Turbomole’s “universal” auxiliary basis sets^69^ were used for the RI-J approximation^70,71^ to the computation of Coulomb integrals. Unless stated otherwise, the SCF energy convergence criterion was set to 1 × 10^–7^ Hartree.

The computational requirements of ωLH25tdE are identical to those of the underlying ωLH22t RSLH. Timing comparisons of ωLH22t with other functionals have been provided in ref 15. For example, self-consistent energy calculations are a factor of 2–3 more costly within the seminumerical integration scheme than for a normal local hybrid without range separation or for a global hybrid, while scaling with system or basis-set size is the same.

## 4 Results and Discussion

### 4.1 Performance of GMTKN55

As summarized in Table 1 with a detailed overview of all functionals compared here and illustrated in Figure 1, the newly developed ωLH25tdE-D4 functional improves significantly over its predecessor, ωLH23tdE-D4, in all GMTKN55 subcategories as well as for the final WTMAD-2 value. The latter is decreased from 3.76 kcal/mol with ωLH23tdE-D4 to 2.64 kcal/mol with ωLH25tdE-D4, indicating an enhanced accuracy in capturing dynamic correlation effects. This value is below the post-BHandHLYP value obtained with Becke’s sophisticated and difficult to routinely use B22plus rung 4 functional^72^ and clearly below any previously reported self-consistent result for a rung 4 functional. Indeed, the 3.10 kcal/mol for our recent LH24n-D4 with neural-network LMF and B97c power-series expansion for correlation^27^ comes closest. A WTMAD-2 of 2.64 kcal/mol is in the range of what is usually considered rung 5 territory. This holds in addition to the further advantages that ωLH25tdE-D4 has regarding the zero-sum game (see further below). The largest improvements pertain to the iso & large as well as the inter- and intramolecular NCI subcategories. As noted in the Introduction, we find the B97c correlation functional in particular to be better suited to work with the D4 dispersion corrections than the previous B95c model. ωLH25tdE-D4 performs better in almost all categories than previous rung 4 functionals, except for LH24n-D4 for intramolecular NCIs or DM21 for the basic and small subcategory (the post-BHandHLYP intermolecular NCI value for B22plus is also lower).

**Figure 1:**
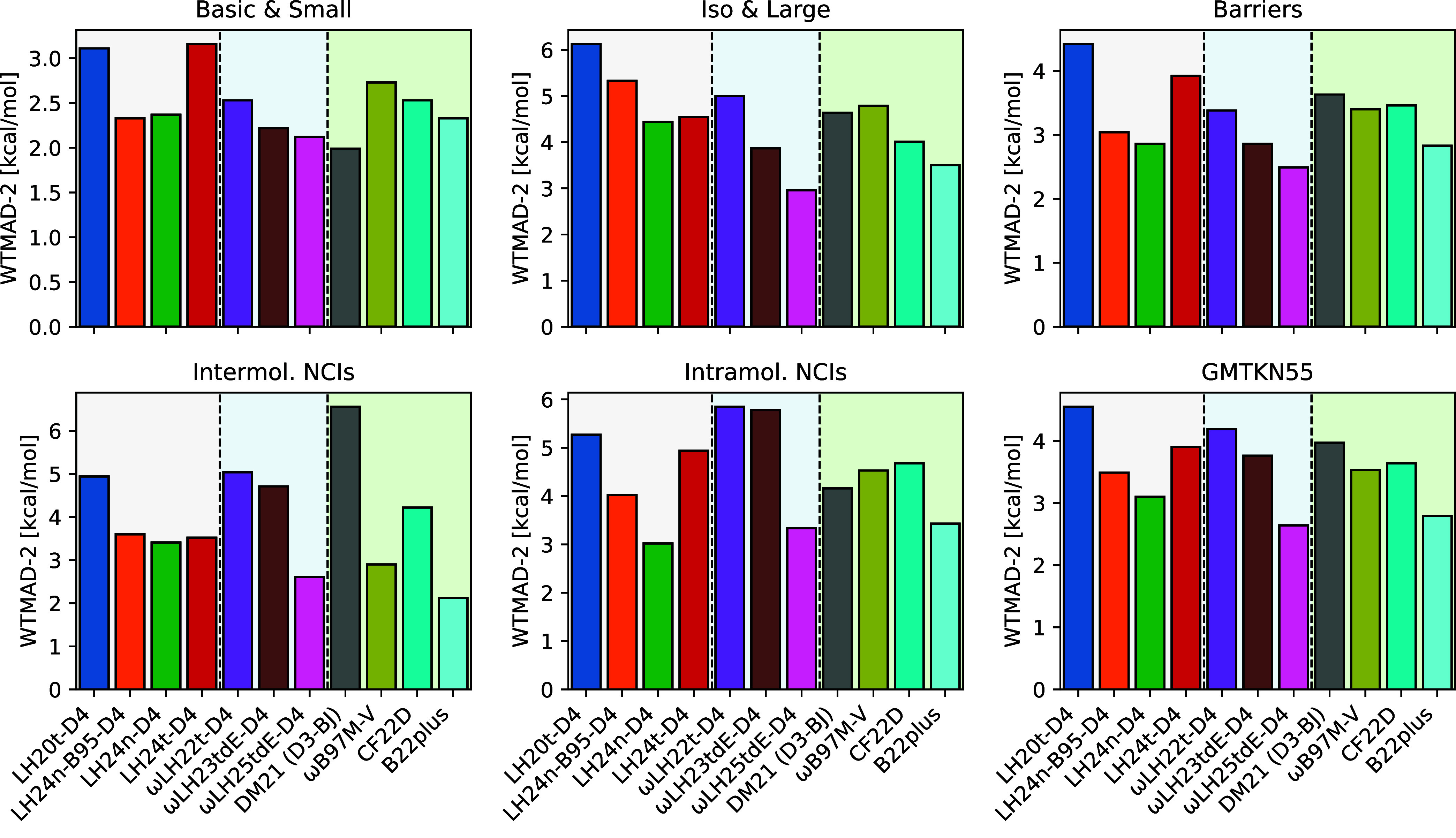
Comparison of WTMAD-2 values across the usual five GMTKN55 subcategories and overall data set performance for various LH, RSLH, and other functionals trained on large data sets (DM21,^23^ ωB97M-V,^73,74^ CF22D,^75^ and B22plus^72^—the latter post-BHLYP for 54 out of 55 subsets).

**Table 1:** Overview of all LH/RSLH Functionals Considered in This Work Including Their Components, Training Data, and GMTKN55 WTMAD-2 Values^a^

functional	ω-EXX^b^	LMF	correlation	sc	DEC	WTMAD-2	training data^c^
ωLH25tdE-D4	yes	t-LMF	B97c	yes	yes	2.64	refit on GMTKN55
ωLH23tdE-D4	yes	t-LMF	B95c	yes	yes	3.76	G2-1/BH76/DISS10
LH24n-D4	no	n-LMF	B97c	no	no	3.10	W4-17/BH76/GMTKN55
LH24n-B95-D4	no	n-LMF	B95c	no	no	3.49	W4-17/BH76
LH20t-D4	no	t-LMF	B95c	no	no	4.55	G2-1/BH76
scLH22t-D4	no	t-LMF	B95c	yes	no	4.46	G2-1/BH76/DISS10
ωLH22t-D4	yes	t-LMF	B95c	no	no	4.17	W4-08/BH76
*ωLH22t(B97c)* ^d^	yes	t-LMF	B97c	no	no	3.00	refit on GMTKN55
*ωLH22t(B95c)* ^d^	yes	t-LMF	B95c	no	no	3.50	refit on GMTKN55
*LH20t(B97c)* ^d^	no	t-LMF	B97c	no	no	3.90	refit on GMTKN55

a Columns sc and DEC indicate the presence of strong-correlation and delocalization-error corrections, respectively. A name in italic font indicates a non-self-consistently computed WTMAD-2 value.

b Yes, full long-range exact exchange; no, no range separation.

c Primary data sets or optimization targets; “refit” indicates a reoptimization of linear parameters.

d Taking the orbitals of the original published functional indicated to the left, only the linear parameters of the dynamical correlation functional (B95c or B97c) have been reoptimized post-SCF on GMTKN55 for these three functionals.

Like its predecessor ωLH23tdE-D4, ωLH25tdE-D4 features sc- and DEC correction terms (see also below). To assess the effect of the improved B97c dynamical correlation in the absence of such correction terms, in comparison to B95c-based ωLH22t, we also replaced B95c in the latter and reparameterized (only) the linear parameters in the same way as for ωLH25tdE-D4. In post-ωLH22t calculations, this gives a non-self-consistent WTMAD-2 value of 3.0 kcal/mol compared to the reported self-consistent value of 4.17 kcal/mol for ωLH22t-D4 (see *ωLH22t­(B97c)* in Table 1). This clearly suggests that the use of less flexible B95c correlation and the choice of small W4-08/BH76 training sets to obtain ωLH22t were suboptimal in this context. Retaining B95c but reparameterizing the linear parameters of this “uncorrected” functional for the full GMTKN55 suite in the presence of D4 corrections provides a post-SCF value of ca. 3.5 kcal/mol (see *ωLH22t­(B95c)* in Table 1), suggesting that the optimization procedure and the choice of B97c vs B95c play a similarly large role in the final improvements. It should be noted that optimization-related improvements are larger than those reported for LH24n-D4 vs LH24n-B95-D4 in the absence of full long-range EXX corrections.^27^ Previously, we have also tested^27^ a replacement of the original B95c correlation (optimized for G2-1/BH76) of LH20t-D4 by GMTKN55-optimized B97c correlation. The post-LH20t result in this case is 3.9 kcal/mol compared to the reported self-consistent LH20t-D4 value of 4.55 kcal/mol (see *LH22t­(B97c)* in Table 1). Just comparing the final post-SCF values with a t-LMF with or without range separation (without sc- or DEC corrections), therefore, suggests that full long-range EXX contributions enable a ca. 0.9 kcal/mol lower WTMAD-2 value (3.0 kcal/mol vs 3.9 kcal/mol).

Comparing the best post-SCF value without the sc- and DEC corrections to the LMF (3.0 kcal/mol with reparameterized B97c) to the overall self-consistent value for ωLH25tdE-D4 (2.64 kcal/mol) also provides us with an estimate of the moderate but notable effects of these terms for GMTKN55 performance (only somewhat less than the lowering of −0.4 kcal/mol from ωLH22t-D4 to ωLH23tdE-D4 ^10^). This is interesting as one usually considers GMTKN55 to be dominated by weakly correlated systems. Part of the improvement arises from the DEC terms, which favorably affect, for example, barriers and other subsets that encode delocalization errors. However, sc corrections have been found previously to also make a small but non-negligible contribution, for example, −0.1 kcal/mol with scLH22t-D4 vs LH20t-D4 ^12^ or −0.16 kcal/mol with ωLH23tdE vs ωLH23td.^10^ This arises from only a relatively small number of systems, which can sometimes see considerable individual change. For example, ωLH22t-D4 makes an error of about 35 kcal/mol for the atomization energy of the C_2_ molecule at its equilibrium structure, while ωLH25tdE-D4 reduces this error to approximately 4 kcal/mol.

### 4.2 Analysis of Possible Overfitting and Transferability

Given that we have now trained the linear parameters for the full GMTKN55 set and then evaluated the results for this set, it is important to examine any likelihood of overfitting. While training on the entire data set and evaluating on it is now a standard practice in many publications related to the development of DFAs, we performed additional validation to ensure the robustness of our approach. Overfitting is not expected to be a major issue, as we optimize only 12 parameters for a very flexible functional based on well-defined physical quantities such as the reduced density gradient, local kinetic-energy density, and so on. We nevertheless conducted additional training experiments where a randomly selected percentage of reactions from each of the 55 test set subsets within GMTKN55 was used as the training set, while the remaining reactions in each subset served as the validation set.

For each partitioning, we minimized WTMAD-2 using the selected training reactions, while keeping the rest for validation. We also report the total WTMAD-2 levels across all reactions. Since GMTKN55 still consists of a relatively limited representation of 1505 reactions, and it is known that a small fraction of reactions is particularly difficult to model, we repeated the entire procedure for 10 different random splits to perform a statistical analysis. The results of this analysis for different percentage splits are shown in Figure 2.

**Figure 2:**
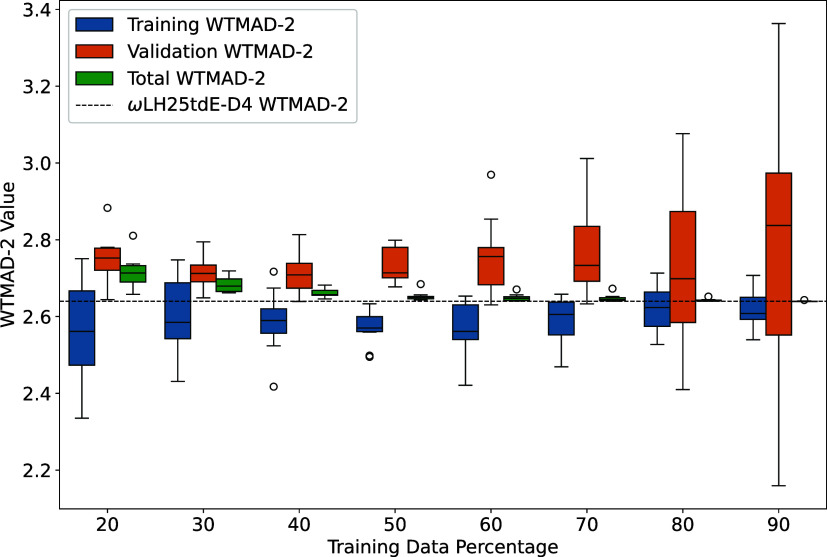
Dependence of WTMAD-2 on the percentage of reactions used for training. The box plots illustrate the distribution of WTMAD-2 values across 10 independent random splits for each training fraction. The central line in each box represents the median, while the interquartile range (IQR) highlights the variability. Outliers correspond to particularly difficult reaction sets. The dashed horizontal line indicates the final self-consistent value for ωLH25tdE-D4.

We did not consider a 10% training fraction since some of the 55 subsets contain fewer than 10 reactions, which would result in an empty training set for those cases. The results indicate that for training fractions of 20% and 30%, the trained WTMAD-2 values exhibit considerable variance, likely due to the presence of difficult reactions in only some of the training sets. However, even with only 20% of reactions used in training, the total WTMAD-2 remains around 2.7 kcal/mol on average and exceeds 2.8 kcal/mol in only one instance. Increasing the training percentage leads to better agreement with the fully trained, self-consistently computed WTMAD-2 value of 2.64 kcal/mol. Independent from the specific training data selection, convergence toward the fully trained result is obtained when 50% of reactions are included in the training set. This suggests that using half of GMTKN55 provides sufficient representation, at least for the overall WTMAD-2 metric. Interestingly, increasing the training fraction beyond 50% leads to a larger validation WTMAD-2 and greater variance. We suspect that this arises from difficult reactions dominating the statistics of the smaller validation set. In any case, the stable total WTMAD-2 values across different splits indicate essentially no overfitting.

### 4.3 Evaluation of ωLH25tdE-D4 for the Large, Automatically Generated W4-11RE Reaction-Energy Test Set

The overall GMTKN55 performance is significantly influenced by NCIs. While reaction energies are also well represented within the test suite, larger sets of just regular reaction energies exist. An example is the W4-11RE set of more than 11000 high-level reaction energies that can be generated easily from the underlying W4-11 atomization-energy set of only 140 molecules.^76^ It has been argued that the performance of functionals for such atomization energies does not predict their performance for the derived reaction energies. We have therefore evaluated ωLH25tdE-D4 also for the W4-11RE set and show results in Table 2, in comparison to other rung 4 functionals that are top performers for GMTKN55. Of the functionals evaluated, ωLH25tdE-D4 and ωLH23tdE-D4 are the only ones with an MAD below 3 kcal/mol. To the best of our knowledge, these are the best rung 4 performances for this test set, and only some rung 5 functionals still perform somewhat better.^77,78^

**Table 2:** Performance of Selected Rung 4 Functionals for the Mean Absolute Deviation (MAD) in kcal/mol of the Automatically Generated W4-11RE Reaction-Energy Test Set^a^

	(kcal/mol)
ωLH25tdE-D4	2.65
ωLH23tdE-D4	2.84
LH24n-D4	3.10
ωB97M-V	3.25
LH20t-D4	3.63
ωB97X-D	3.71
ωB97X-V	3.73
ωLH22t-D4	3.74
M05-2X	4.68

a All data generated with identical settings in this work.

### 4.4 Evaluation of ωLH25tdE-D4 for Organometallic Transition-Metal Reaction Energies and Barriers

We have previously found LH20t-D4 and ωLH22t-D4 to be among the top-performing functionals for three test sets on organometallic transition-metal reactivity, that is, the MOR41 set of closed-shell reaction energies,^45^ the ROST61 set of open-shell reaction energies,^46^ and the modified MOBH28 subset^51^ of the MOBH35 reaction-barrier set.^47–50^ Tables S11 and S12 summarize the results obtained for these same sets for ωLH25tdE-D4 and for ωLH23tdE-D4. The overall MADs of ωLH25tdE-D4 for MOR41, ROST61, and MOBH28 are close to the corresponding values for the above-mentioned ωLH22-D4, indicating that neither the sc- and DEC corrections nor the modified B97c dynamical correlation have a large impact on these tests. Only for ROST61 is the MAD slightly increased (2.87 kcal/mol compared to 2.44 kcal/mol for ωLH23tdE-D4). This can be contrasted to values of 3.87 and 3.38 kcal/mol for the recent LH24n-D4 and LH24n-B95-D4 with neural-network LMFs, where transferability is less favorable, while B97c correlation apparently also deteriorates the results very slightly compared to the original B95c correlation.

### 4.5 Shifting the Zero-Sum Game: Strong Correlation and Delocalization Error Cases

The attractiveness of ωLH23tdE and related scRSLHs has been that they reduce significantly static correlation errors as indicated, for example, by spin-restricted bond dissociation curves^10^ and by the reduction of spin contamination in certain open-shell transition-metal complexes.^14^ At the same time, delocalization errors remain small (see also below). As we largely changed only the dynamical correlation contributions on going from ωLH23tdE-D4 to ωLH25tdE-D4, we expect that these advantages in escaping the zero-sum game are retained. This is supported by the plot of the MAEs of the DISS10 set of spin-restricted bond-dissociation asymptotes for 10 second- and third-period main-group diatomics against GMTKN55 WTMAD-2 values in Figure 3. As has been known,^7,9^ most functionals lie roughly on a line with a negative slope, which indicates the zero-sum game. scRSLHs such as ωLH23tdE or DM21 have been previously shown to move this Pareto front to the lower left of the graph. Obviously, ωLH25tdE-D4 is also very effective in this context: the DISS10 value of 32 kcal/mol is very similar to that of ωLH23tdE-D4 (31 kcal/mol), while the lower WTMAD-2 value moves the point clearly to the left from either ωLH23tdE-D4 or DM21. At present, none of the human-designed functionals achieves the essentially zero DISS10 MAD of the deep-neural-network DM21, which has been trained specifically for such measures (in the form of fractional spin errors).^23^ We note in passing that using a neural-network LMF in the recent LH24n-D4 moves its entry clearly to the left of LH20t-D4, while a slightly larger DISS10 value keeps it close to the original Pareto front, indicating the zero-sum game. This clearly points to the need to incorporate terms for strong correlations.

**Figure 3:**
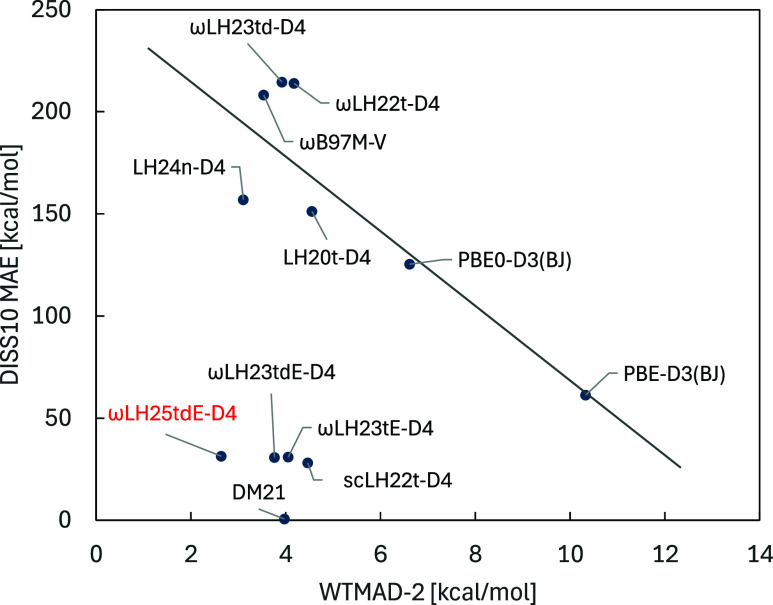
Evaluation of the zero-sum game for selected (RS)­LH functionals (PBE, PBE0, and ωB97M-V are given as examples of GGA, GH, and RSH functionals, respectively). The MAE for DISS10 is a measure of strong correlation errors, while GMTKN55 WTMAD-2 is a measure of performance for weakly correlated situations.

The dissociation curves of noble-gas radical cation dimers are prototypical tests of delocalization errors.^3^ We evaluated ωLH25tdE for Ne_2_^+^ and Ar_2_^+^ radical cations and provided the curves in Figure S1 in the Supporting Information. The asymptote is slightly below but similar to that of ωLH23tdE. Unlike functionals without full long-range EXX admixture (scLH22t is given as an example of an LH), the local maximum and subsequent decrease toward the dissociation asymptote is avoided by ωLH25tdE, as well as by ωLH23tdE or ωLH22t.

We recently showed^14^ that the DEC- and sc corrections of scRSLHs allow a significant reduction of the spin contamination problem of certain open-shell transition-metal complexes in comparison to uncorrected RSLHs or other hybrids with appreciable EXX admixtures. We have looked at the prototypical MnO_3_ complex used in that context. Just like ωLH23tdE, ωLH25tdE achieves a substantial improvement, reducing the ⟨*S* ^2^ ⟩ value from 0.926 for ωLH22t to 0.754 for this doublet system. This is accompanied by an improved dipolar ^55^ Mn HFC *A*_dip_ = 96 MHz (cf. 97 MHz for ωLH23tdE and exp. 81 MHz) compared to overestimated values with global, local, range-separated, or range-separated local hybrids without such correction terms (cf., e.g.,^14^ PBE0: *A*_dip_ = 138 MHz, ⟨*S* ^2^ ⟩ ≈ 1.0; ωLH22t: *A*_dip_ = 134 MHz).

### 4.6 Retaining the Correct Asymptotic Exchange-Correlation Potential: Quasiparticle Energies

One clear advantage of RSLHs such as ωLH22t is their correct long-range asymptotic potential combined with the added flexibility of a short-range position-dependent EXX admixture. It has been shown that this provides very good TDDFT results for charge-transfer excitations^15^ as well as an excellent description of ionization potentials (IPs), electron affinities (EAs), and fundamental gaps for a wide variety of systems based on using the frontier orbitals as quasiparticle energies invoking Koopmans’ theorem (within a generalized Kohn-Sham framework).^16^ Notably, this is achieved without a system-dependent tuning of the single-range separation parameter ω in contrast to the widely used “optimal tuning strategy”. Gratifyingly, scRSLHs like ωLH23tdE largely retain these advantages^10^ in spite of the sc correction terms that interrupt the full long-range EXX admixture in spatial regions where strong correlations are detected (see *f*_*FR*_(**r**) Δ*e*_LR,σ_(**r**) contribution in eq 1).

Figure 4 shows a compact graphical representation of the performance of ωLH25tdE in this context; full numerical data are provided in Tables S4–S10 in Supporting Information. The systems investigated here are (a) electron affinities of atoms from periods 2 and 3 computed from the HOMO energy of the anion,^58^ (b) IPs, EAs, and gaps of a series of oligoacences (sizes *n* = 1–6)^79^ from HOMO and LUMO energies of the neutral system, and (c) the same for another series of important acceptor molecules in organic photovoltaics.^80^ ωLH25tdE performs somewhat worse than ωLH23tdE in all cases but remains close to the former functional, with the possible exception of category (c), where the deterioration is somewhat larger.

**Figure 4:**
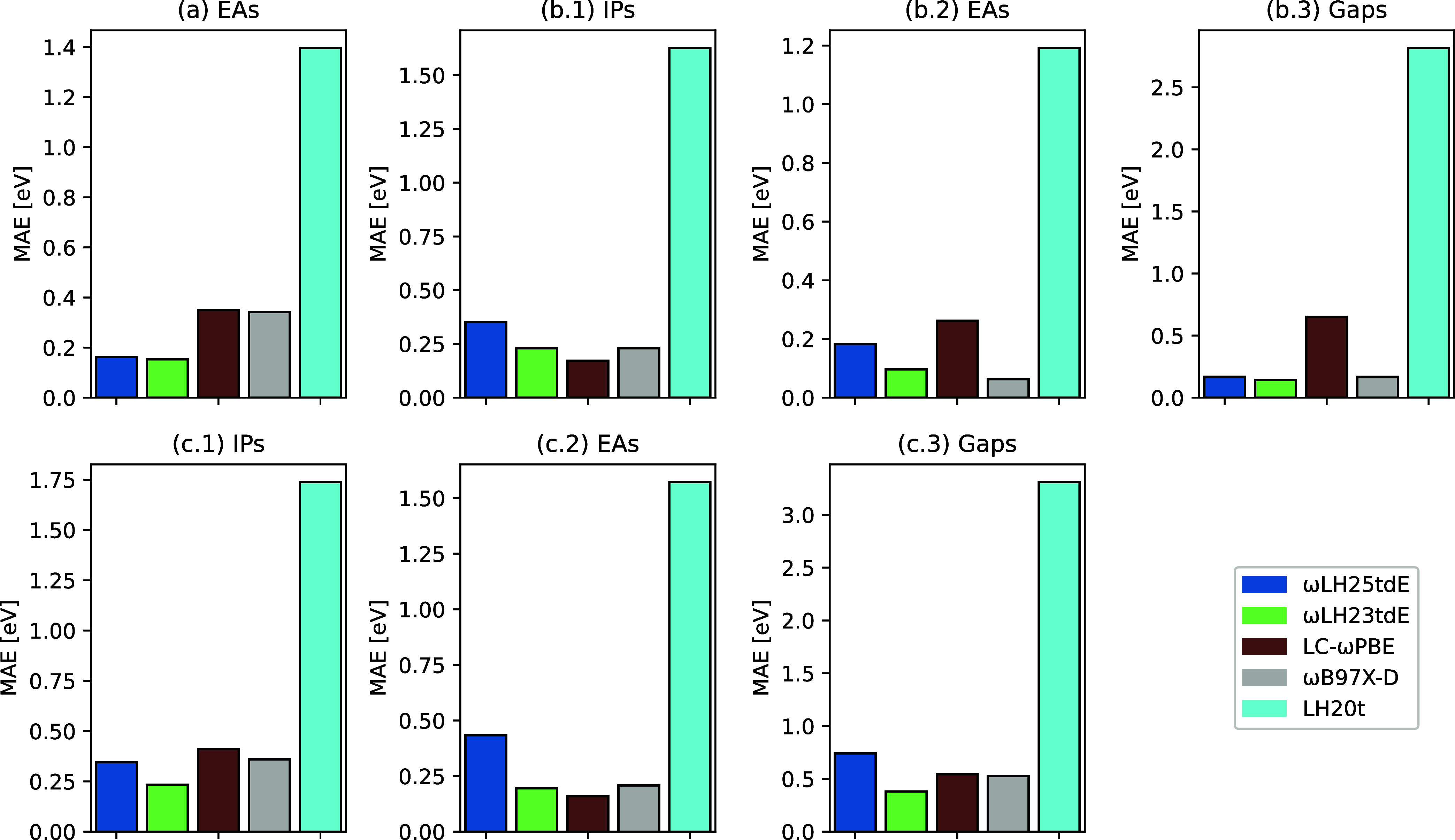
Bar plot of MAEs in eV for a variety of test sets of IPs, EAs, and fundamental gaps computed with different functionals from HOMO and LUMO energies. (a) EAs of atomic anions from the HOMO energy of the anion, cf. ref 58; (b) oligoacene IPs, EAs, and gaps from HOMO and LUMO energies of the neutral molecule, ref 79; (c) IPs, EAs, and gaps from HOMO and LUMO energies of the neutral molecule for a series of chromophores from ref 80.

Closer analysis suggests that the interplay between using B97c (instead of B95c in ωLH23tdE) and optimization of the correlation functional for full GMTKN55 leads to a slight upward shift of the LUMO energies, which in this case is detrimental for EAs and gaps. While this can probably be improved upon by adding properties dependent on the frontier-orbital energies to the training, we have not attempted to do this here. In any case, ωLH25tdE retains in large parts the advantages of long-range EXX admixture in comparison to functionals without long-range corrections (see, e.g., ref 16, and references therein).

## 5 Conclusions

In recent work, it has been shown that functionals like ωLH23tdE allow us to substantially shift the zero-sum game between minimizing delocalization and static correlation errors in modern hybrid functionals. That is, stretched bonds and systems with static correlation can be improved upon while also maintaining small self-interaction errors and excellent asymptotic XC potentials. In this work, we have explored to what extent we can improve the already excellent performance of weakly correlated systems as represented by the large GMTKN55 suite, by replacing B95c correlation with a more flexible B97c-type power-series expansion optimized in conjunction with D4 dispersion corrections.

When augmented by D4 corrections, the resulting ωLH25tdE functional achieves a striking WTMAD-2 value of 2.64 kcal/mol for GMTKN55. This is an improvement of more than 1 kcal/mol over ωLH23tdE-D4 and represents the lowest value of any rung 4 functional so far, in fact, moving us into what is typically assumed “double-hybrid territory.” At the same time, ωLH25tdE still provides substantial improvements in the description of spin-restricted bond dissociation and of spin densities in open-shell transition-metal complexes over standard functionals while also showing excellent HOMO and LUMO energies for use in quasiparticle descriptions of ionization potentials, electron affinities, and band gaps for many chromophores. We also expect good performance for various excitation classes in TDDFT calculations (a corresponding TDDFT implementation of such functionals is underway).

The present results emphasize not only the outstanding role that the exact exchange-energy density can play in the construction of modern density functionals that move us outside the usual zero-sum game but also the importance of other parts of the functional, such as dynamical correlation.

## Supplementary Material



## Notes

The complete set of fitted ωLH25tdE-D4 and D4-dispersion parameters, a table of full GMTKN55 deviations, WTMAD-2 values for GMTKN55 and subcategories, and spin-unrestricted Ne_2_^+^/Ar_2_^+^ dissociation curves as cases dependent on delocalization errors; several tables with CCSD­(T)-benchmarked data for ionization potentials, electron affinities, and fundamental gaps of oligoacenes and other diverse acceptor molecules are provided, as well as tables with detailed data for MOR41, ROST61, and MOBH28 organometallic reaction energies and barrier heights, including statistical evaluations (PDF)
