# Supplementary material for: “Beyond-Zero-Sum” Range-Separated Local Hybrid Functional with Improved Dynamical Correlation
Source: J Chem Theory Comput. 2025 Jul 18;21(15):7419–29. doi: 10.1021/acs.jctc.5c00699 (PMC12355696; doi:10.1021/acs.jctc.5c00699)
Supplement: Supplementary file 1 [file ct5c00699_si_001.pdf]

Supplementary Information: "Beyond-zero-sum" range-separated local hybrid functional with improved dynamical correlation

Artur Wodyński* and Martin Kaupp*

Technische Universität Berlin, Institut für Chemie, Theoretische Chemie/Quantenchemie, Sekr. C7, Straße des 17. Juni 135, D-10623, Berlin, Germany

E-mail: artur.wodynski@tu-berlin.de; martin.kaupp@tu-berlin.de

Table S1. Optimized parameters of ωLH25tdE and the associated D4 dispersion corrections.[a]

	parameter	Value
$g(\mathbf{r})$	a	0.587
	h	12
	e	**0.917541**
$q_{\mathrm{AC}}^{erf}(\mathbf{r})$	b	3.6380410
	c	0.073
range separation	ω	0.233
pig2 CF	f_1	-2.49722
	f_2	1.31151
	β	0.001
B97c	c_{opp}	0.00198
	$c_{\sigma\sigma}$	0.047712
	$d_{\mathrm{opp},0}$	**1.122846**
	$d_{\mathrm{opp},1}$	**-2.383741**
	$d_{\mathrm{opp},2}$	**4.615269**
	$d_{\mathrm{opp},3}$	**-3.117071**
	$d_{\sigma\sigma,0}$	**0.293130**
	$d_{\sigma\sigma,1}$	**-0.835147**
	$d_{\sigma\sigma,2}$	**1.007257**
	$d_{\sigma\sigma,3}$	**-0.451557**
D4	s_8	**0.04824492**
	a_1	**0.3848141**
	a_2	**3.87795235**

[a]Values of parameters optimized in this work are given in bold print. The others have been retained from ωLH23tdE.[1]

Table S2. Comparison of selected functionals (with D4 corrections) for the MAD of the GMTKN55 subsets (in kcal/mol).

	ωLH25tdE-D4	ωLH23tdE-D4[1]	LH24n-D4[2]
ACONF	0.03	0.09	0.08
ADIM6	0.11	0.30	0.12
AHB21	0.36	0.43	0.25
AL2X6	1.89	0.66	1.07
ALK8	3.73	1.28	1.53
ALKBDE10	3.12	3.36	3.91
AMINO20x4	0.20	0.28	0.14
BH76	1.00	1.07	1.20
BH76RC	1.25	1.20	0.96
BHDIV10	0.77	0.92	0.85
BHPERI	0.67	0.88	0.99
BHROT27	0.32	0.41	0.32
BSR36	0.34	0.61	1.99
BUT14DIOL	0.06	0.17	0.07
C60ISO	1.02	1.04	5.27
CARBHB12	0.26	0.25	0.50
CDIE20	0.38	0.38	0.55
CHB6	0.92	0.51	0.55
DARC	0.64	1.62	0.95
DC13	5.18	8.51	4.07
DIPCS10	7.55	5.04	6.13
FH51	1.21	1.30	1.66
G21EA	1.57	1.31	1.78
G21IP	3.26	2.87	3.25
G2RC	2.00	2.93	2.79
HAL59	0.27	0.47	0.35
HEAVY28	0.13	0.26	0.28
HEAVYSB11	1.01	0.72	0.97
ICONF	0.24	0.25	0.18
IDISP	0.87	1.60	1.12
IL16	1.41	1.73	1.20
INV24	1.07	1.19	1.06
ISO34	0.95	1.33	0.71
ISOL24	1.82	2.30	1.41
MB1643	14.22	11.56	20.25
MCONF	0.13	0.41	0.11
NBPRC	1.11	0.98	1.57
PA26	1.09	1.51	1.91
PArel	0.48	0.72	0.73
PCONF21	0.21	0.48	0.20
PNICO23	0.18	0.18	0.30
PX13	1.71	1.93	1.21
RC21	1.52	2.15	1.62
RG18	0.08	0.23	0.06
RSE43	0.34	0.46	0.44
S22	0.20	0.29	0.17
S66	0.16	0.16	0.12
SCONF	0.14	0.37	0.15
SIE4x4	6.06	4.54	6.90
TAUT15	0.49	0.69	0.59
UPU23	0.74	0.68	0.83
W4-11	2.89	1.94	3.61
WATER27	1.27	1.45	0.91
WCPT18	1.08	1.29	1.60
YBDE18	1.24	1.20	1.89

Table S3. Comparison of selected dispersion-corrected functionals for the total WTMAD-2 value and subcategory values of GMTKN55 (in kcal/mol).

	basic & small	iso & large	barriers	intermol. NCIs	intramol. NCIs	GMTKN55
LH20t-D4[2,3]	3.11	6.13	4.42	4.94	5.27	4.55
LH24n-D4[2]	2.37	4.44	2.86	3.41	3.02	3.10
LH24t-D4[2]	3.16	4.55	3.92	3.52	4.94	3.90
ωLH22t-D4[4]	2.53	5.00	3.38	5.04	5.85	4.19
ωLH23tdE-D4[1]	2.22	3.87	2.86	4.71	5.78	3.76
ωLH25tdE-D4	2.12	2.96	2.49	2.61	3.34	2.64
DM21 (D3-BJ)[5]	1.99	4.64	3.63	6.56	4.16	3.97
ωB97M-V[6,7]	2.73	4.79	3.40	2.90	4.53	3.53
CF22D[8]	2.53	4.01	3.46	4.22	4.68	3.64
B22plus[9,a]	2.33	3.50	2.83	2.12	3.43	2.79

[a] Non-self-consistent results based on BHandHLYP orbitals on 54 out of 55 subsets.

Figure S1. Comparison of spin-unrestricted bond-dissociation curves for the Ne_2^+ and Ar_2^+ radical cations with selected functionals.

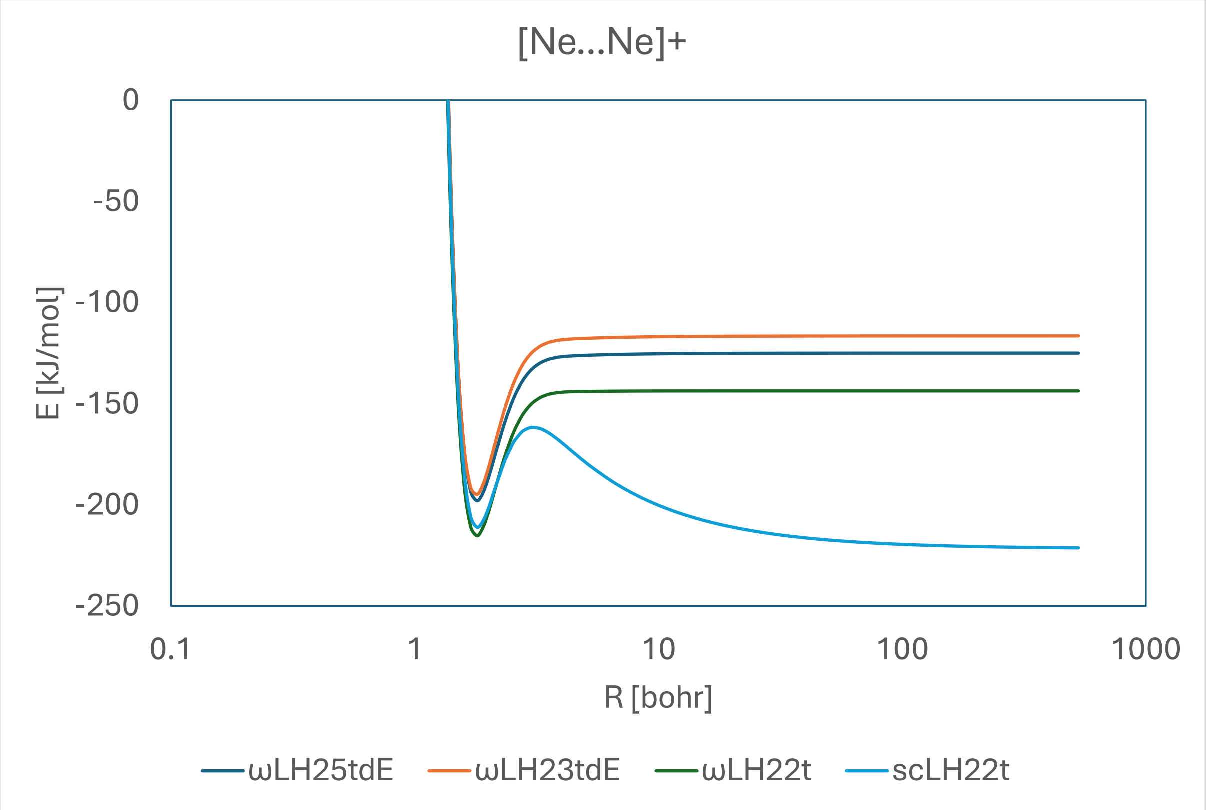

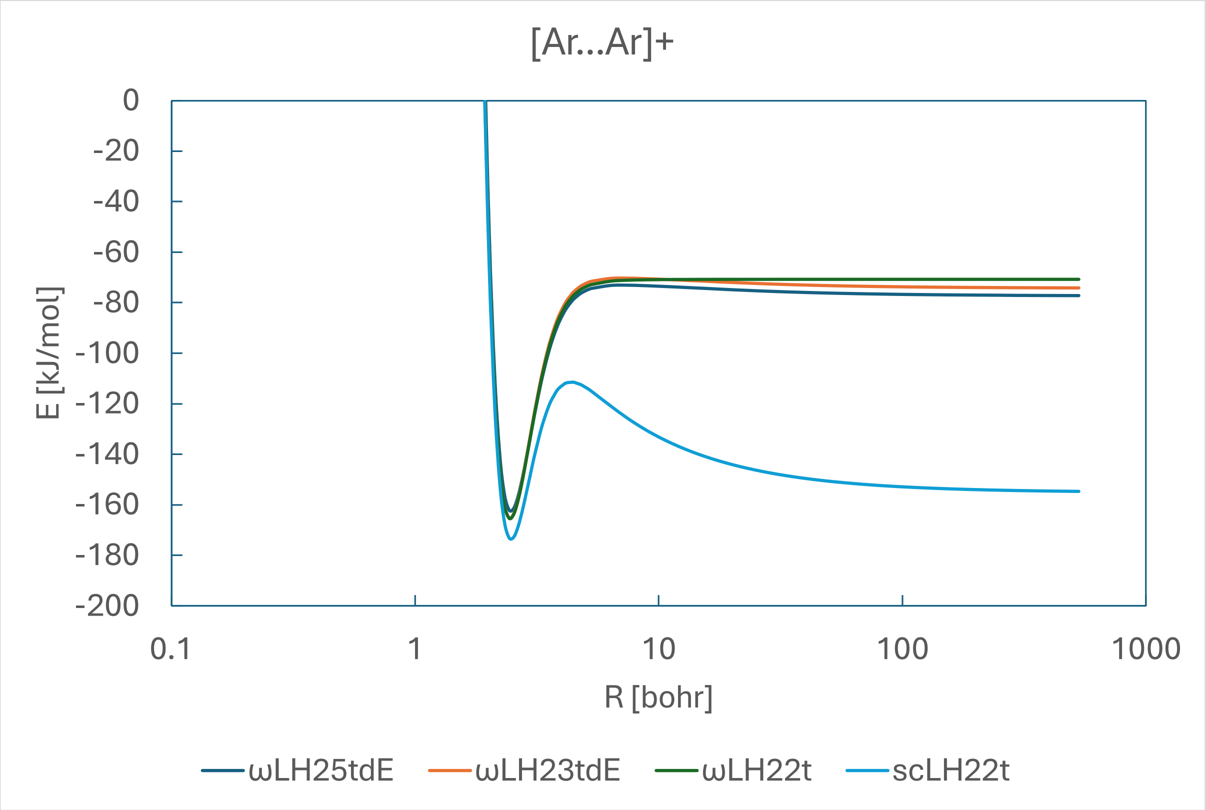

Table S4. Electron affinities in eV of atomic anions from the negative of the HOMO energy of the anion, using aug-pc-∞ basis sets.

	Exp	ωLH25tdE	ωLH23tdE[1]	LC-ωPBE[10]	ωB97X-D[10]	LH20t[10]
Li-	0.62	0.62	0.63	0.79	0.70	0.03
B-	0.28	0.47	0.49	0.86	0.36	-0.79
C-	1.26	1.28	1.35	1.68	0.89	-0.42
O-	1.46	1.47	1.55	1.70	0.87	-0.24
F-	3.40	2.70	2.82	3.11	2.24	1.14
Na-	0.55	0.58	0.60	0.76	0.66	-0.09
Al.-	0.43	0.45	0.47	0.87	0.47	-0.58
Si-	1.39	1.21	1.26	1.81	1.14	-0.12
P-	0.75	0.79	0.77	1.26	0.68	-0.51
S -	2.08	1.93	1.97	2.46	1.75	0.45
Cl-	3.61	3.17	3.25	3.75	2.93	1.60
	MAE	0.16	0.15	0.35	0.34	1.40
	MSE	-0.11	-0.06	0.29	-0.29	-1.40
	AMax	0.70	0.58	0.58	1.16	2.26

Table S5. Ionization potentials of oligoacenes in eV with different functionals from the negative of the HOMO energy (cc-pVTZ basis) in comparison to CCSD(T) reference data.

n	CCSD(T)	ωLH25tdE	ωLH23tdE[1]	LC-ωPBE[10]	ωB97X-D[10]	LH20t[10]
1	9.44	9.02	9.14	9.36	9.02	7.47
2	8.24	7.88	8.00	8.15	7.94	6.48
3	7.47	7.15	7.27	7.32	7.26	5.86
4	6.95	6.65	6.77	6.77	6.80	5.44
5	6.57	6.29	6.41	6.38	6.47	5.15
6	6.43	6.01	6.13	6.09	6.23	4.94
	MAE	0.35	0.23	0.17	0.23	1.63
	MSE	-0.35	-0.23	-0.17	-0.23	-1.63
	AMAX	0.42	0.30	0.34	0.42	1.97

Table S6. Electron Afinities of oligoacenes in eV with different functionals from the negative of the HOMO energy (cc-pVTZ basis) in comparison to CCSD(T) reference data.

n	CCSD(T)	ωLH25tdE	ωLH23tdE[1]	LC-ωPBE[10]	ωB97X-D[10]	LH20t[10]
1	-1.53	-1.85	-1.77	-1.94	-1.63	-0.29
2	-0.48	-0.69	-0.60	-0.80	-0.52	0.79
3	0.28	0.10	0.20	0.03	0.23	1.50
4	0.82	0.65	0.75	0.59	0.75	1.99
5	1.20	1.05	1.15	1.00	1.12	2.32
6	1.47	1.39	1.49	1.31	1.43	2.60
	MAE	0.18	0.10	0.26	0.06	1.19
	MSE	-0.18	-0.09	-0.26	-0.06	1.19
	AMAX	0.32	0.24	0.41	0.10	1.27

Table S7. Fundamental gaps of oligoacenes in eV with different functionals from the negative of the HOMO energy (cc-pVTZ basis) in comparison to CCSD(T) reference data.

n	CCSD(T)	ωLH25tdE	ωLH23tdE[1]	LC-ωPBE[10]	ωB97X-D[10]	LH20t[10]
1	10.97	10.87	10.92	11.75	10.65	7.77
2	8.72	8.56	8.60	9.44	8.46	5.69
3	7.19	7.05	7.07	7.86	7.03	4.35
4	6.13	5.99	6.01	6.78	6.05	3.46
5	5.37	5.24	5.25	6.00	5.35	2.83
6	4.96	4.62	4.64	5.42	4.80	2.34
MAE		0.17	0.14	0.65	0.17	2.82
MSE		-0.17	-0.14	0.65	-0.17	-2.82
AMAX		0.34	0.32	0.78	0.32	3.20

Table S8. Ionization potentials of a diverse set of acceptor molecules from Ref. 11 in eV from the negative of the HOMO energy, in comparison with CCSD(T) reference data from Ref. 12.

	CCSD(T)	ωLH25tdE	ωLH23tdE[1]	LC-ωPBE[10]	ωB97X-D[10]	LH20t[10]
Anthracene	7.52	7.19	7.31	7.90	7.29	5.91
Acridine	8.04	7.71	7.83	8.44	7.80	6.40
Phenazine	8.47	8.15	8.27	8.90	8.23	6.81
Azulene	7.55	7.18	7.30	7.90	7.30	5.90
Benzoquinone	10.27	10.13	10.25	10.74	9.88	8.49
Naphtalenedione	9.88	9.67	9.80	10.45	9.69	8.19
Dichlone	9.99	9.66	9.78	10.44	9.64	8.24
F4-Benzoquinone	11.14	10.60	10.73	11.38	10.52	9.15
Cl4-Benzoquinone	10.25	9.93	10.05	10.71	9.89	8.53
Nitrobenzene	10.19	9.88	10.01	10.66	9.88	8.39
F4-Benzenedicarbonitrile	10.76	10.39	10.52	11.16	10.41	9.04
Dinitrobenzonitrile	11.15	10.91	11.04	11.67	10.87	9.47
Nitrobenzonitrile	10.62	10.33	10.46	11.10	10.32	8.90
Benzonitrile	9.93	9.55	9.68	10.30	9.53	8.07
Fumaronitrile	11.48	10.77	10.89	11.55	10.67	9.29
m-DCNB	10.45	10.10	10.22	10.85	10.08	8.65
TCNE	11.99	11.42	11.54	12.19	11.35	10.01
TCNQ	9.57	9.19	9.31	9.96	9.27	8.07
Maleic Anhydride	11.33	10.99	11.12	11.57	10.68	9.37
Phthalimide	10.55	9.85	9.97	11.04	9.83	8.33
Phtalic Anhydride	10.08	10.28	10.40	10.61	10.27	8.80
Cl4-Isobenzofurandione	10.05	9.89	10.01	10.63	9.89	8.55
NDCA	9.14	8.90	9.02	9.59	8.93	7.54
Boron-Dipyrromethene	8.07	7.89	8.00	8.60	8.01	6.64
	MAE	0.35	0.23	0.41	0.36	1.74
	MSE	-0.33	-0.21	0.41	-0.34	-1.74
	AMAX	0.71	0.59	0.58	0.81	2.22

Table S9. Electon affinities of a diverse set of acceptor molecules from Ref. 11 in eV from the negative of the LUMO energy, in comparison with CCSD(T) reference data from Ref. 12.

	CCSD(T)	ωLH25tdE	ωLH23tdE[1]	LC-ωPBE[10]	ωB97X-D[10]	LH20t[10]
Anthracene	0.33	0.61	0.31	0.08	0.31	1.59
Acridine	0.69	0.71	0.66	0.44	0.66	1.95
Phenazine	1.11	1.02	1.11	0.89	1.10	2.40
Azulene	0.54	0.45	0.53	0.31	0.52	1.84
Benzoquinone	1.55	1.81	1.87	1.39	1.96	3.38
Naphtalenedione	1.47	1.49	1.57	1.28	1.65	3.04
Dichlone	1.92	1.90	1.98	1.65	1.98	3.40
F4-Benzoquinone	2.29	2.63	2.73	2.33	2.69	4.20
Cl4-Benzoquinone	2.48	2.59	2.68	2.28	2.63	4.09
Nitrobenzene	0.54	1.35	0.87	0.47	0.86	2.30
F4-Benzenedicarbonitrile	1.62	1.76	1.85	1.57	1.81	3.27
Dinitrobenzonitrile	1.76	1.99	2.06	1.65	2.02	3.49
Nitrobenzonitrile	1.30	1.66	1.63	1.28	1.62	3.05
Benzonitrile	-0.21	1.09	-0.12	-0.37	-0.04	1.31
Fumaronitrile	0.98	1.70	1.38	1.07	1.48	2.83
m-DCNB	0.61	1.27	0.72	0.45	0.77	2.14
TCNE	3.05	3.38	3.47	3.17	3.45	4.86
TCNQ	3.33	3.57	3.66	3.40	3.60	4.83
Maleic Anhydride	1.01	1.82	1.39	1.03	1.48	2.95
Phthalimide	0.87	1.13	0.68	0.70	0.75	2.19
Phtalic Anhydride	0.63	1.33	0.97	0.40	1.04	2.49
Cl4-Isobenzofurandione	1.68	1.62	1.71	1.36	1.66	3.15
NDCA	1.26	1.39	1.28	1.04	1.30	2.64
Boron-Dipyrromethene	1.67	4.12	1.68	1.45	1.61	2.84
	MAE	0.43	0.20	0.16	0.21	1.57
	MSE	0.41	0.17	-0.13	0.18	1.57
	AMAX	2.45	0.44	0.32	0.50	1.94

Table S10. Fundamental gaps of a diverse set of acceptor molecules from Ref. 11 in eV from the negative of the HOMO and LUMO energies, in comparison with CCSD(T) reference data from Ref. 12.

	CCSD(T)	ωLH25tdE	ωLH23tdE[1]	LC-ωPBE[10]	ωB97X-D[10]	LH20t[10]
Anthracene	7.19	6.58	7.00	7.82	6.98	4.33
Acridine	7.35	7.00	7.16	8.00	7.14	4.45
Phenazine	7.36	7.13	7.16	8.01	7.12	4.41
Azulene	7.01	6.73	6.77	7.59	6.78	4.07
Benzoquinone	8.72	8.33	8.38	9.35	7.93	5.12
Naphtalenedione	8.41	8.19	8.23	9.17	8.04	5.15
Dichlone	8.07	7.76	7.80	8.79	7.66	4.83
F4-Benzoquinone	8.85	7.96	8.01	9.05	7.83	4.95
Cl4-Benzoquinone	7.77	7.34	7.38	8.43	7.27	4.44
Nitrobenzene	9.65	8.53	9.14	10.18	9.02	6.09
F4-Benzenedicarbonitrile	9.14	8.63	8.67	9.59	8.60	5.77
Dinitrobenzonitrile	9.39	8.92	8.98	10.02	8.86	5.98
Nitrobenzonitrile	9.32	8.68	8.83	9.83	8.70	5.84
Benzonitrile	10.14	8.46	9.80	10.67	9.57	6.76
Fumaronitrile	10.50	9.07	9.51	10.48	9.20	6.45
m-DCNB	9.84	8.83	9.51	10.39	9.31	6.50
TCNE	8.94	8.03	8.07	9.03	7.90	5.16
TCNQ	6.24	5.62	5.65	6.56	5.67	3.24
Maleic Anhydride	10.32	9.17	9.74	10.54	9.21	6.43
Phthalimide	9.68	8.72	9.29	10.34	9.08	6.14
Phtalic Anhydride	9.45	8.94	9.43	10.20	9.23	6.31
Cl4-Isobenzofurandione	8.37	8.28	8.30	9.26	8.23	5.40
NDCA	7.88	7.51	7.74	8.55	7.63	4.90
Boron-Dipyrromethene	6.40	3.76	6.32	7.16	6.40	3.80
MAE		0.74	0.38	0.54	0.53	3.31
MSE		-0.74	-0.38	0.54	-0.53	-3.31
AMAX		2.64	0.99	0.89	1.30	4.05

Table S11. Comparison of MOR41 reaction-energy results and statistical data (in kcal/mol) for LH20t-D4, LH24n-D4, LH24n-B95-D4, ωLH25tdE-D4, ωLH23tdE-D4 and ωLH22t-D4

Reaction	Reference	LH20t-D4[13]	LH24n-D4[2]	LH24n-B95-D4[2]	ωLH25tdE-D4	ωLH23tdE-D4	ωLH22t-D4[4]
1	-43.10	-42.30	-39.46	-38.11	-40.42	-41.97	-41.97
2	-46.60	-44.12	-38.12	-36.65	-43.57	-45.19	-45.19
3	-27.60	-23.41	-15.86	-14.23	-23.21	-24.35	-24.35
4	-62.50	-64.08	-59.53	-61.06	-62.54	-62.15	-61.93
5	3.70	-1.96	0.94	1.31	4.05	3.95	1.52
6	-23.20	-17.58	-18.07	-17.28	-18.64	-20.27	-20.06
7	-16.20	-12.73	-15.01	-14.12	-11.31	-13.52	-15.54
8	-17.20	-8.21	-12.30	-12.65	-12.97	-12.74	-13.21
9	-18.70	-5.52	-10.74	-12.25	-17.39	-14.70	-11.23
10	-22.60	-16.63	-13.99	-13.63	-20.73	-20.70	-17.99
11	27.00	29.29	34.03	33.89	30.00	30.42	30.98
12	-29.80	-29.60	-27.02	-25.45	-27.76	-29.56	-29.55
13	-43.20	-43.49	-40.58	-39.66	-41.78	-43.24	-43.04
14	-52.00	-51.61	-43.67	-42.24	-47.84	-51.43	-52.24
15	-4.10	-4.79	-13.24	-17.72	-6.70	-5.37	-5.35
16	-39.80	-39.73	-40.10	-38.50	-39.25	-40.86	-40.77
17	-16.10	-14.27	-15.61	-15.46	-15.24	-16.08	-16.71
18	-34.20	-32.73	-31.10	-31.45	-29.39	-28.81	-31.83
19	-40.10	-39.27	-37.42	-38.05	-36.09	-35.63	-38.52
20	-30.20	-30.32	-29.34	-30.00	-27.91	-26.98	-29.84
21	-15.10	-16.93	-17.65	-17.05	-16.32	-14.87	-13.50
22	-35.90	-34.97	-30.73	-30.42	-23.87	-23.10	-29.71
23	-55.00	-52.26	-49.45	-50.47	-46.14	-45.37	-50.64
24	-41.60	-41.54	-40.55	-40.08	-38.93	-41.20	-42.48
25	-45.90	-45.82	-44.85	-43.99	-43.54	-46.28	-46.36
26	-36.40	-35.20	-36.71	-38.80	-37.67	-34.01	-34.09
27	-21.80	-23.43	-23.97	-24.46	-24.18	-21.85	-21.83
28	-36.30	-35.98	-37.16	-38.73	-38.16	-35.06	-35.04
29	-28.30	-26.17	-28.71	-29.96	-28.85	-25.77	-25.59
30	-14.00	-10.94	-15.09	-16.83	-15.61	-9.89	-9.32
31	-29.90	-26.69	-28.49	-30.65	-29.66	-25.90	-25.90
32	-1.80	-2.08	-0.56	-0.33	-1.35	-1.94	-1.98
33	-10.70	-7.84	-10.13	-12.15	-13.05	-9.45	-8.39
34	-25.60	-24.61	-23.73	-25.38	-26.75	-24.22	-23.37
35	-30.90	-29.92	-29.05	-30.81	-30.82	-28.64	-28.33
36	-39.80	-36.88	-34.57	-36.66	-34.33	-32.44	-34.37
37	-14.00	-16.31	-16.24	-18.12	-16.12	-10.68	-10.58
38	-64.40	-62.55	-63.15	-66.55	-64.51	-59.11	-59.11
39	-63.90	-64.19	-60.99	-60.70	-64.00	-64.74	-64.69
40	-65.80	-67.12	-66.31	-66.39	-66.01	-67.02	-66.96
41	-3.20	-2.21	-2.07	-3.16	-4.30	-2.21	-0.79

		LH20t-D4	LH24n-B95-D4	LH24n-D4	ωLH25tdE-D4	ωLH23tdE	ωLH22t-D4
MAE		2.25	3.30	3.65	2.47	2.53	2.30
RMSD		3.44	4.44	5.01	3.47	3.71	2.96
MSE		1.47	2.21	1.75	1.56	2.29	1.88
MaxE(−)		-5.66	-9.14	-13.62	-2.60	-1.27	-2.18
MaxE(+)		13.18	11.74	13.37	12.03	12.80	7.47
STDEV		3.11	3.85	4.68	3.09	2.90	2.30

Table S12. Comparison of ROST61 reaction-energy results and statistical data (in kcal/mol) for LH20t-D4, LH24n-D4, LH24n-B95-D4, ωLH25tdE-D4, ωLH23tdE-D4 and ωLH22t-D4

	ref	LH20t-D4[13]	LH24n-D4[2]	LH24n-B95-D4[2]	ωLH25tdE-D4	ωLH23tdE-D4	ωLH22t-D4[4]
1	-40.05	-29.52	-30.34	-29.74	-28.12	-25.83	-27.72
2	-37.71	-46.09	-48.55	-46.61	-47.49	-48.68	-47.77
3	-12.59	-14.13	-14.21	-14.25	-12.27	-12.59	-13.36
4	-17.04	-16.33	-12.78	-14.44	-15.01	-16.62	-16.81
5	-10.66	-10.28	-11.92	-12.24	-10.50	-10.62	-10.65
6	-12.66	-8.93	-7.27	-6.27	-7.83	-6.84	-7.56
7	-14.73	-14.74	-14.76	-15.34	-14.86	-15.55	-15.48
8	-10.51	-9.08	-10.91	-10.20	-9.61	-9.16	-9.05
9	-12.76	-13.62	-13.11	-12.63	-11.52	-10.58	-12.53
10	-21.20	-26.01	-25.62	-26.05	-24.23	-25.10	-26.28
11	-8.44	-12.39	-12.51	-13.42	-12.70	-14.51	-13.76
12	-2.79	-1.99	-1.19	-1.27	-1.65	-1.70	-1.66
13	-178.96	-184.56	-198.16	-195.97	-188.51	-190.21	-185.15
14	-42.33	-43.84	-45.75	-44.83	-44.07	-45.07	-44.78
15	-7.17	-8.61	-7.91	-7.35	-9.17	-8.49	-8.29
16	-193.43	-191.40	-183.49	-186.49	-185.98	-192.03	-192.10
17	-200.10	-201.05	-193.56	-196.11	-193.39	-200.35	-200.86
18	-203.09	-202.72	-196.49	-198.84	-196.59	-202.61	-202.61
19	-4.94	1.98	6.50	5.69	0.60	1.36	1.80
20	-27.02	-30.24	-28.75	-29.47	-28.39	-28	-27.37
21	-25.09	-26.29	-29.42	-28.99	-28.06	-27.24	-27.14
22	-46.70	-47.76	-47.54	-47.60	-45.60	-47.28	-46.55
23	-115.35	-108.01	-109.42	-108.82	-109.58	-110.49	-110.17
24	-46.37	-45.16	-48.61	-48.50	-46.30	-47.62	-47.47
25	-19.39	-14.85	-11.87	-10.08	-14.81	-13.79	-14.33
26	-47.73	-50.03	-49.55	-50.04	-49.12	-49.95	-50.10
27	-25.86	-23.06	-23.58	-23.24	-24.54	-24.52	-24.42
28	-28.77	-25.91	-26.75	-26.26	-27.20	-27.04	-27.23
29	-11.13	-11.89	-11.77	-11.81	-12.11	-11.81	-11.76
30	-141.32	-141.58	-142.18	-141.31	-142.27	-141.29	-141.49
31	-63.73	-62.74	-58.11	-60.55	-58.58	-63.26	-63.13
32	-49.75	-50.14	-49.74	-49.29	-51.14	-51.30	-51.55
33	-10.64	-19.14	-20.47	-18.63	-21.63	-23.50	-22.53
34	-0.65	-0.81	-0.67	-0.82	-0.49	-0.77	-0.78
35	-5.46	-14.40	-15.12	-14.36	-15.64	-18.49	-17.54
36	-49.04	-48.18	-42.27	-42.93	-48.54	-47.20	-47.5
37	-40.62	-40.39	-39.66	-39.32	-40.01	-39.71	-39.65
38	-27.70	-28.56	-37.73	-36.29	-27.53	-28.32	-28.64
39	-44.99	-46.19	-49.02	-47.94	-46.46	-45.40	-45.21
40	-24.51	-25.65	-23.00	-24.16	-23.53	-24.65	-24.83
41	-5.96	-6.68	-6.46	-6.33	-6.58	-6.5	-6.54
42	-26.96	-26.69	-25.54	-26.71	-23.28	-24.15	-24.44
43	-80.58	-85.88	-85.07	-85.08	-84.62	-85.31	-85.79
44	-66.15	-71.15	-70.43	-70.73	-69.75	-71.06	-71.37
45	-42.83	-45.31	-47.56	-46.37	-44.53	-43.47	-44.00
46	-33.27	-32.91	-31.09	-31.93	-29.29	-31.98	-33.03
47	-58.47	-58.55	-55.91	-57.44	-54.44	-57.63	-57.75
48	-4.24	-3.74	-8.72	-7.67	-5.70	-4.95	-5.39
49	-35.26	-34.10	-34.27	-33.60	-34.67	-34.98	-34.98
50	-2.74	-3.68	-0.50	-1.40	-1.12	-2.59	-2.46
51	-29.77	-30.99	-34.39	-33.06	-32.33	-30.06	-30.22
52	-42.72	-46.54	-49.55	-47.82	-43.08	-44.09	-48.00
53	-30.78	-29.59	-30.34	-29.85	-29.67	-30.66	-30.64
54	-12.33	-12.79	-7.89	-8.30	-11.84	-11.74	-10.67
55	-4.61	-4.56	-4.59	-4.30	-4.83	-4.67	-4.79
56	-13.65	-13.92	-14.00	-13.90	-14.03	-13.58	-13.35
57	-31.89	-32.28	-28.45	-29.94	-30.02	-31.95	-31.77
58	-39.30	-40.76	-38.00	-39.38	-38.38	-40.04	-39.87
59	-29.89	-31.90	-30.12	-30.75	-25.96	-26.91	-31.40
60	-66.00	-65.31	-61.53	-63.03	-61.52	-65.06	-65.26
61	-69.48	-70.11	-71.11	-71.15	-69.96	-70.40	-70.49

		LH20t-D4[13]	LH24n-D4[2]	LH24n-B95-D4[2]	ωLH25tdE-D4	ωLH23tdE-D4	ωLH22t-D4[4]
MAE		2.23	3.87	3.38	2.87	2.44	2.32
RMSD		3.35	5.35	4.75	4.11	4.21	3.83
MSE		-0.52	-0.21	-0.26	0.32	-0.47	-0.60
MaxE(–)		-8.94	-19.20	-17.01	-10.99	-13.03	-12.08
MaxE(+)		10.53	11.44	10.63	11.93	14.22	12.33
STDEV		3.34	5.39	4.78	4.13	4.22	3.82

Table S13. Comparison of MOBH28 reaction-barrier results (in kcal/mol) for LH20t-D4, LH24n-D4, LH24n-B95-D4, ωLH25tdE-D4, ωLH23tdE-D4 and ωLH22t-D4

barrier	ref	LH20t-D4[13]	LH24n-D4[2]	LH24n-B95-D4[2]	ωLH25tdE-D4	ωLH23tdE-D4	ωLH22t-D4[4]
1_fwd	26.20	26.49	28.37	27.31	27.55	26.94	27.08
2_fwd	5.71	6.71	6.93	7.14	6.83	6.38	6.66
3_fwd	0.92	0.90	1.18	1.11	1.16	0.97	1.25
4_fwd	1.36	0.63	0.91	0.92	1.11	0.79	0.71
5_fwd	4.63	5.87	5.70	5.22	4.83	4.65	4.91
6_fwd	15.76	15.14	15.51	15.09	15.31	14.19	14.82
7_fwd	27.59	26.51	27.33	26.11	27.74	27.86	29.01
8_fwd	34.57	33.31	31.27	32.13	33.89	37.03	34.34
10_fwd	-4.29	0.86	-1.42	0.15	-0.82	0.65	-2.24
11_fwd	29.49	29.05	27.28	28.40	24.83	29.40	28.22
12_fwd	5.50	4.86	4.65	4.66	4.68	4.12	4.60
13_fwd	20.65	18.34	23.48	21.23	19.08	14.12	20.12
14_fwd	10.10	10.65	10.30	10.21	10.11	9.83	9.93
15_fwd	20.66	17.95	20.24	18.42	20.24	18.07	18.14
16_fwd	35.45	34.98	33.14	33.12	34.18	33.93	33.25
21_fwd	8.41	8.58	9.79	8.44	11.00	8.70	8.41
22_fwd	13.84	12.35	14.71	13.49	15.11	12.39	14.03
23_fwd	29.45	28.53	31.97	31.07	31.58	29.94	31.19
26_fwd	25.83	20.80	22.03	22.10	16.59	19.95	25.32
27_fwd	14.05	12.82	12.63	12.39	13.11	13.47	13.07
28_fwd	30.18	31.04	29.52	29.11	24.11	24.51	30.54
29_fwd	14.72	15.52	14.19	14.06	14.60	14.00	14.55
30_fwd	9.79	11.58	10.64	9.87	9.75	8.98	9.94
31_fwd	2.91	7.65	6.68	6.50	3.38	1.29	5.68
32_fwd	20.18	20.90	22.14	21.29	21.38	20.14	19.69
33_fwd	1.05	0.01	1.14	0.85	3.60	2.79	-0.15
34_fwd	29.16	27.63	26.57	25.95	28.85	29.28	27.63
35_fwd	17.28	16.09	15.84	16.00	16.90	17.82	17.14
1_rev	14.02	14.70	18.05	16.00	18.11	14.91	15.57
2_rev	22.25	20.00	23.39	22.52	22.62	22.64	21.70
3_rev	26.92	32.83	32.00	31.41	31.07	32.18	32.63
4_rev	8.25	11.71	12.13	11.61	12.08	12.44	12.95
5_rev	22.60	22.01	21.85	21.69	24.82	24.66	22.09
6_rev	14.61	12.95	12.57	12.07	12.63	11.99	12.78
7_rev	18.58	16.35	18.42	17.47	18.90	17.19	18.72
8_rev	31.82	29.34	23.63	24.33	29.53	29.91	31.10
10_rev	8.22	2.40	6.40	5.91	6.70	4.41	5.03
11_rev	82.34	80.96	84.56	83.26	84.65	84.94	83.33
12_rev	37.18	40.16	38.19	37.08	38.83	36.54	37.28
13_rev	47.99	48.83	52.70	50.54	48.70	46.28	52.09
14_rev	14.37	13.83	15.26	14.45	14.87	14.00	14.07
15_rev	74.98	81.15	78.83	80.97	79.49	84.70	85.81
16_rev	53.77	57.41	57.12	58.23	55.40	58.72	59.64
21_rev	8.41	8.58	9.79	8.45	11.00	8.71	8.41
22_rev	27.01	25.09	28.58	25.84	29.36	25.55	27.62
23_rev	20.35	20.47	22.58	22.33	22.56	22.28	23.49
26_rev	0.11	0.07	0.14	0.20	-0.13	0.11	0.01
27_rev	2.29	1.97	1.81	1.56	2.89	2.14	2.22
28_rev	15.52	15.93	15.87	15.56	16.37	16.36	16.69
29_rev	31.19	29.88	29.60	28.84	30.66	29.86	30.31
30_rev	16.60	17.91	19.39	18.49	18.79	17.96	17.38
31_rev	12.90	11.72	11.85	11.28	12.11	13.62	12.32
32_rev	62.62	69.39	63.83	67.48	63.19	69.41	70.63
33_rev	7.86	10.32	11.43	11.12	7.05	7.23	10.61
34_rev	3.04	4.88	5.39	4.98	3.98	3.51	4.45
35_rev	-2.44	-2.30	-2.08	-2.06	-2.05	-2.59	-1.79

Table S14. Comparison of MOBH28 barrier-set statistical data (in kcal/mol) for LH20t-D4, LH24n-D4, LH24n-B95-D4, ωLH25tdE-D4, ωLH23tdE-D4 and ωLH22t-D4

		LH20t-D4[13]	LH24n-D4[2]	LH24n-B95-D4[2]	ωLH25tdE-D4	ωLH23tdE-D4	ωLH22t-D4[4]
all	MAE	1.76	1.87	1.74	1.63	1.83	1.55
	RMSD	2.45	2.42	2.38	2.35	2.77	2.56
	MSE	0.17	0.56	0.17	0.36	0.13	0.72
	MaxE(−)	-5.82	-8.19	-7.49	-9.24	-6.53	-3.19
	MaxE(+)	6.77	5.08	5.99	4.51	9.72	10.83
	STDEV	2.47	2.38	2.39	2.35	2.79	2.48
only ΔE_{fwd}	MAE	1.43	1.52	1.38	1.57	1.56	0.91
	RMSD	1.97	1.89	1.81	2.58	2.42	1.19
	MSE	-0.19	0.06	-0.32	-0.37	-0.68	-0.12
	MaxE(−)	-5.03	-3.80	-3.73	-9.24	-6.53	-2.52
	MaxE(+)	5.15	3.77	4.44	3.47	4.94	2.77
	STDEV	2.00	1.92	1.82	2.60	2.37	1.20
only ΔE_{rev}	2.09	2.22	2.10	1.68	2.09	2.19	
	RMSD	2.85	2.86	2.83	2.10	3.07	3.43
	MSE	0.54	1.07	0.65	1.10	0.94	1.56
	MaxE(−)	-5.82	-8.19	-7.49	-2.29	-3.81	-3.19
	MaxE(+)	6.77	5.08	5.99	4.51	9.72	10.83
	STDEV	2.85	2.70	2.80	1.82	2.98	3.11

References

(1) Fürst, S.; Kaupp, M.; Wodyński, A. Range-separated local hybrid functionals with small fractional-charge and fractional-spin errors: escaping the zero-sum game of DFT functionals. *J. Chem. Theory Comput.* **2023**, *19*, 8639–8653.

(2) Wodyński, A.; Glodny, K.; Kaupp, M. Data-Driven Improvement of Local Hybrid Functionals: Neural-Network-Based Local Mixing Functions and Power-Series Correlation Functionals. *J. Chem. Theory Comput.* **2025**, *21*, 762–775.

(3) Haasler, M.; Maier, T. M.; Grotjahn, R.; Gückel, S.; Arbuznikov, A. V.; Kaupp, M. A local hybrid functional with wide applicability and good balance between (de)localization and left-right correlation. *J. Chem. Theory Comput.* **2020**, *16*, 5645–5657.

(4) Fürst, S.; Haasler, M.; Grotjahn, R.; Kaupp, M. Full Implementation, Optimization, and Evaluation of a Range-Separated Local Hybrid Functional with Wide Accuracy for Ground and Excited States. *J. Chem. Theory Comput.* **2023**, *19*, 488–502.

(5) Kirkpatrick, J.; McMorrow, B.; Turban, D. H. P.; Gaunt, A. L.; Spencer, J. S.; Matthews, A. G. D. G.; Obika, A.; Thiry, L.; Fortunato, M.; Pfau, D.; Castellanos, L. R.; Petersen, S.; Nelson, A. W. R.; Kohli, P.; Mori-Sánchez, P.; Hassabis, D.; Cohen, A. J. Pushing the frontiers of density functionals by solving the fractional electron problem. *Science* **2021**, *374*, 1385–1389.

(6) Mardirossian, N.; Head-Gordon, M. ωB97M-V: A combinatorially optimized, range-separated hybrid, meta-GGA density functional with VV10 nonlocal correlation. *J. Chem. Phys.* **2016**, *144*, 214110.

(7) Najibi, A.; Goerigk, L. The nonlocal kernel in van der Waals density functionals as an additive correction: An extensive analysis with special emphasis on the B97M-V and ωB97M-V approaches. *J. Chem. Theory Comput.* **2018**, *14*, 5725–5738.

(8) Liu, Y.; Zhang, C.; Liu, Z.; Truhlar, D. G.; Wang, Y.; He, X. Supervised learning of a chemistry functional with damped dispersion. *Nat. Comput. Sci.* **2023**, *3*, 48–58.

(9) Becke, A. D. Density-functional theory vs density-functional fits: The best of both. *J. Chem. Phys.* **2022**, *157*, 234102.

(10) Fürst, S.; Kaupp, M. Accurate Ionization Potentials, Electron Affinities, and Band Gaps from the ωLH22t Range-Separated Local Hybrid Functional: No Tuning Required. *J. Chem. Theory Comput.* **2023**, *19*, 3146–3158.

(11) Gallandi, L.; Marom, N.; Rinke, P.; Körzdörfer, T. Accurate ionization potentials and electron affinities of acceptor molecules II: non-empirically tuned long-range corrected hybrid functionals. *J. Chem. Theory Comput.* **2016**, *12*, 605–614.

(12) Richard, R. M.; Marshall, M. S.; Dolgounitcheva, O.; Ortiz, J. V.; Bredas, J.-L.; Marom, N.; Sherrill, C. D. Accurate ionization potentials and electron affinities of acceptor molecules I. Reference data at the CCSD(T) complete basis set limit. *J. Chem. Theory Comput.* **2016**, *12*, 595–604.

(13) Grotjahn, R.; Kaupp, M. A Look at Real-World Transition-Metal Thermochemistry and Kinetics with Local Hybrid Functionals. *Isr. J. Chem.* **2022**, *63*, e202200021.
